# Sex-Specific Associations of Vegetable and Fruit Intake Categories with Depressive Symptoms Modified by Weight-Adjusted Waist Index Among Chinese Older Adults

**DOI:** 10.3390/nu18121941

**Published:** 2026-06-16

**Authors:** Liang Huang, Zixuan Hong, Mingming Liu, Dongmei Zhang

**Affiliations:** 1 Department of Toxicology, School of Public Health, Anhui Medical University, Hefei 231200, China; 2 Department of Health Services Management, School of Health Services Management, Anhui Medical University, Hefei 231200, China

**Keywords:** vegetable and fruit intake categories, depressive symptoms, weight-adjusted waist index, central adiposity, sex differences, cross-sectional study, older adults, China

## Abstract

Background: Depressive symptoms are a growing public health concern among aging populations. However, whether the association between vegetable and fruit intake and depressive symptoms varies by central adiposity and sex remains unclear. Objectives: This study aimed to examine whether vegetable and fruit intake categories are associated with depressive symptoms, and to evaluate whether the Weight-Adjusted Waist Index (WWI) and sex jointly modify these associations among older adults in Anhui Province, China. Methods: This cross-sectional study employed multistage stratified sampling across four cities in Anhui Province, China, from July to September 2019. Data on sociodemographic characteristics, WWI, weekly vegetable and fruit intake frequency, and depressive symptoms were collected from 5737 participants. Multivariable binary logistic regression models were employed to examine the associations, with analyses stratified by sex. Interaction analyses were conducted to evaluate the modifying roles of sex and WWI. Results: Among 5737 participants, the prevalence of depressive symptoms was 32.46%. After full adjustment, the V+/F− category was associated with higher odds of depressive symptoms in women (AOR = 1.25, 95% CI: 1.04–1.49). An interaction between vegetable and fruit intake categories and WWI levels was observed (*p*_interaction_ = 0.048). In stratified analyses, significant associations were observed among Q1 females with V+/F− and V−/F− categories and among Q3 males with the V−/F− category, whereas most other subgroup associations were not statistically significant after adjustment. Conclusions: The associations between vegetable and fruit intake and depressive symptoms may vary by metabolic status, as indexed by WWI, and by sex among older Chinese adults. Observed associations were more pronounced in females with low WWI and males with moderate WWI, and weaker among those with the highest WWI. These findings are exploratory and hypothesis-generating given the cross-sectional design and borderline interaction significance. Future longitudinal and intervention studies are needed to confirm these relationships and clarify the joint roles of dietary intake and central adiposity in late-life depressive symptoms.

## 1. Introduction

Depression is a prevalent and disabling mental disorder that contributes substantially to the global burden of disease, particularly among older adults [1,2,3]. According to the Global Burden of Disease Study 2023, depression affects approximately 4% of the global population and remains a leading cause of disability worldwide [3]. In China, the lifetime prevalence of depressive disorders has been estimated at 6.8%, with depression contributing substantially to disability and suicide burden nationwide [4,5,6]. Given the rapid population aging occurring in China, identifying modifiable factors associated with depressive symptoms in later life has become an important public health priority.

Nutritional intake is increasingly recognized as an important factor associated with mental health outcomes, including depression, anxiety, and suicidal ideation [7,8]. Evidence from prospective studies suggests that adherence to healthy dietary patterns is linked to a lower risk of depressive symptoms [9]. For example, higher adherence to the Mediterranean diet has been consistently associated with reduced depressive symptom risk, while higher fruit and vegetable consumption has been associated with a lower likelihood of depressive symptoms [10,11,12]. Among older adults, vegetable- and fruit-rich dietary patterns have also been associated with better cognitive function and lower frailty risk [13,14]. However, findings across studies remain heterogeneous, and some associations are attenuated after adjustment for baseline depressive symptoms and other confounders [15]. These inconsistencies suggest that the relationship between diet and depressive symptoms may depend on individual biological and metabolic characteristics [16].

China is currently experiencing a rapid dietary transition from traditional dietary patterns toward diets characterized by higher consumption of fat, sugar, and animal-source foods, accompanied by insufficient fruit intake [17,18]. Although national nutrition initiatives continue to promote increased consumption of vegetables and fruits, a distinctive pattern of relatively high vegetable intake but inadequate fruit intake remains common among older Chinese adults [19,20]. Understanding how such dietary patterns relate to depressive symptoms may therefore have particular relevance in the Chinese context.

Body composition represents another factor associated with mental health [21,22]. While obesity is generally linked to a higher risk of depressive symptoms, studies among older adults have reported an “obesity paradox,” in which higher body mass index (BMI) is associated with lower depressive symptom burden [23,24,25,26]. Traditional central adiposity indicators such as waist circumference (WC) and waist-to-height ratio (WHtR) are influenced by body size and age-related reductions in stature, further limiting their utility in older adults [27]. Consequently, more precise indicators of central adiposity are needed [28].

The Weight-Adjusted Waist Index (WWI), calculated as waist circumference divided by the square root of body weight, has emerged as a novel anthropometric measure of central adiposity that is relatively independent of body weight [29]. Compared with conventional obesity indicators, WWI may better capture the normal-weight obesity phenotype and age-related changes in body composition [30,31]. Given that central adiposity is closely linked to systemic inflammation, oxidative stress, and metabolic dysregulation, WWI may provide additional insight into the relationship between adiposity and depressive symptoms [32,33]. Nevertheless, evidence regarding the association between WWI and depressive symptoms, particularly in the context of vegetable and fruit intake categories, remains limited.

The present study further conceptualizes WWI not only as an indicator of central adiposity but also as a potential modifier of the association between vegetable and fruit intake and depressive symptoms. These processes may influence how dietary factors relate to mental health [34,35,36]. These relationships may also differ by sex as men and women exhibit distinct fat distribution patterns, metabolic profiles, and hormonal environments [37]. However, few studies have examined whether the associations between vegetable and fruit intake and depressive symptoms vary according to central adiposity status and sex among older adults. We therefore investigated the independent and joint associations of vegetable and fruit intake categories and WWI with depressive symptoms among older adults in Anhui Province, China. We also examined whether these associations differed across WWI levels and between men and women.

## 2. Materials and Methods

### 2.1. Participants

Data were drawn from the Anhui Healthy Longevity Survey (AHLS), a large cross-sectional survey conducted in Anhui Province, China, from July to September 2019 [14,38]. We applied multistage stratified sampling to obtain a geographically diverse study population from Anhui Province. First, four cities—Chuzhou, Xuancheng, Lu’an, and Fuyang—were chosen to ensure regional coverage across the east, south, west, and north of Anhui. Next, a random selection of three to five communities, including both urban and rural settings, was conducted in each city. Inclusion criteria required participants to be at least 60 years of age, with the cognitive and communicative capacity to provide informed consent. Face-to-face interviews were conducted on-site by trained investigators. Of the 6211 participants initially enrolled, we excluded those with incomplete assessments for depression severity (*N* = 32) or missing data for the weight-adjusted waist index (WWI) (*N* = 442). A final sample of 5737 participants was included in the analysis. Ethical approval was granted by the Biomedical Ethics Committee of Anhui Medical University (No. 2020H011). More detailed content about our sampling process has been introduced in our prior publication [39].

### 2.2. Variables and Instruments

#### 2.2.1. Assessment of Depressive Symptoms

Depressive symptoms in participants were evaluated with the Patient Health Questionnaire (PHQ-9). The PHQ-9 is a self-assessment tool used for screening and evaluating the severity of depressive symptoms [40]. The scale comprises nine items corresponding to the core symptoms of depression. The frequency of symptoms over the preceding two weeks was assessed using a 4-point Likert scale (0–3), yielding a cumulative score between 0 and 27. Following the original PHQ-9 severity classification and previous epidemiological studies, the Cronbach’s alpha for the PHQ-9 in the present sample was 0.83, indicating good internal consistency. A score of ≥5 was used to identify individuals with depressive symptoms, representing at least mild symptom severity [41,42].

#### 2.2.2. Vegetable and Fruit Intake Categories

Dietary habits were assessed based on the reported weekly frequency of vegetable (V) and fruit (F) consumption. Participants selected one of four options: “every day”, “4–6 days”, “2–3 days”, or “less than 1 day”. Consistent with previous studies [39,43], consumption on ≥4 days per week was classified as regular intake (V+ or F+), and consumption on <4 days per week as occasional intake (V− or F−). Participants were then categorized into four vegetable and fruit intake categories: V+/F+, V+/F−, V−/F+, and V−/F−.

#### 2.2.3. Weight-Adjusted Waist Index

WWI was calculated as waist circumference (cm) divided by the square root of body weight (kg) [29]. Waist circumference and weight were measured on-site using standardized protocols. WWI was categorized into quartiles (Q1–Q4) based on its distribution in the total study population: Q1 (7.27–10.80), Q2 (10.80–11.33), Q3 (11.33–11.94), and Q4 (11.94–16.08). Quartiles were derived from the pooled study population to maintain a common absolute WWI scale across males and females, thereby facilitating comparisons of the same WWI range between sexes in the stratified and interaction analyses.

#### 2.2.4. Other Variables

To document potential behavioral risk factors for depressive symptoms alongside participants’ sociodemographic profiles, our team administered an original, researcher-designed questionnaire. This tool systematically recorded core variables including age in years, sex, residential location (urban or rural), living arrangements (solitary or cohabiting), marital condition (married or other), and educational attainment (no formal schooling, primary education, or higher). Financial and lifestyle metrics were also gathered simultaneously, namely yearly earnings (<6500 RMB or ≥6500 RMB), smoking status, and drinking status. Clinical characteristics, including chronic diseases, cognitive impairment, and disability in activities of daily living (ADL), were also assessed. Cognitive impairment among respondents was screened using the Chinese version of the Mini-Mental State Examination (MMSE) [20]. Data regarding chronic diseases were collected by presenting respondents with the following query: “Do you suffer from one of the following chronic diseases (hypertension, hyperlipidemia, diabetes, chronic hepatitis, cancer, heart disease, stroke, lung disease, psychiatric disease, etc.)”. Disability in ADL was assessed using the ten-item ADL scale and dichotomized as “Yes” or “No” according to previous studies [39]. Participants with a total ADL score of 100 were classified as having no disability, whereas those scoring <100 were classified as having ADL disability.

### 2.3. Statistical Analysis

Baseline characteristics were summarized as frequencies and percentages (unweighted), with differences across WWI quartiles compared using chi-square tests.

Binary logistic regression models were employed to estimate odds ratios (ORs) and 95% confidence intervals (CIs) for the associations between WWI, vegetable and fruit intake categories, and depressive symptoms. Two models were constructed: Model 1 was unadjusted, while Model 2 was fully adjusted for potential covariates, including age in years, residential location, living arrangements, marital condition, educational attainment, yearly earnings, smoking status, drinking status, chronic diseases, cognitive impairment, disability in ADL, vegetable and fruit intake categories and WWI.

To further explore the complex interrelationships among body composition, lifestyle, and mental health, two interaction analyses were performed:

- WWI × sex, to examine sex-specific associations between WWI and depressive symptoms;
- vegetable and fruit intake categories × WWI, to evaluate whether WWI acts as an effect modifier for the dietary-depressive symptoms link.

Based on the interaction analyses, subsequent stratified analyses were conducted by sex and WWI quartiles, respectively. These analyses were prespecified based on the study hypotheses.

All statistical analyses were performed using SPSS 25.0 (IBM Corp., Armonk, NY, USA). A two-tailed *p* < 0.05 was considered statistically significant.

## 3. Results

### 3.1. Participants Characteristics and Sex-Specific Divergence

A total of 5737 participants (2621 males and 3116 females) were included and categorized into quartiles of the WWI (Table 1 and Table 2). The overall prevalence of depressive symptoms was 32.46%. A marked sex-specific pattern was observed in the distribution of WWI: the majority of males were clustered in the lowest quartile (Q1: 37.50%), whereas most females were in the highest quartile (Q4: 38.77%).

### 3.2. Sex-Specific Patterns of Depressive Symptoms and Intake Categories Across WWI Quartiles

Sex-specific differences were observed in depressive symptoms and vegetable and fruit intake categories across WWI quartiles. Among females, the prevalence of depressive symptoms showed a significant graded increase across WWI quartiles. In contrast, this association lacked statistical significance among male participants (*p* = 0.102).

With respect to vegetable and fruit intake categories, the V+/F− group was the most prevalent across all participants. The distribution of intake categories did not differ significantly across WWI quartiles in either sex (males: *p* = 0.346; females: *p* = 0.087). Among females, the proportion of the V+/F+ group showed a modest decreasing trend across WWI quartiles (Q1: 34.86% vs. Q4: 29.14%).

### 3.3. Distribution of Clinical and Functional Characteristics Across WWI Quartiles

In both sexes, higher WWI was accompanied by a graded increase in multimorbidity and functional impairment. Significant dose–response increases in chronic diseases, ADL disability, and cognitive impairment were observed from Q1 to Q4 (all *p* < 0.001). The prevalence of cognitive impairment in males nearly doubled across the WWI gradient, rising from 22.69% in Q1 to 44.34% in Q4.

### 3.4. Sociodemographic and Lifestyle Factors

Participants in higher WWI quartiles were significantly older (*p* < 0.001 for both sexes). In males, higher WWI was associated with rural residence (*p* = 0.003) and lower yearly earnings (*p* = 0.008), but not with living arrangements, marital condition, or educational attainment. Females in the highest WWI quartile exhibited higher proportions of living alone (*p* = 0.002), being in non-marital status (*p* < 0.001), and having lower educational attainment (67.80% no formal schooling in Q4 vs. 58.82% in Q1, *p* < 0.001).

Regarding lifestyle factors, smoking and drinking rates were substantially higher in males than in females. Specifically, as WWI increased, the proportion of male smokers dropped from 43.95% to 35.85% (*p* = 0.018), and male drinkers decreased from 60.73% to 51.89% (*p* = 0.011). However, no significant trends were observed in females for either smoking (*p* = 0.631) or drinking (*p* = 0.194).

### 3.5. Main Effects and Interaction Analysis

**Figure 1:**
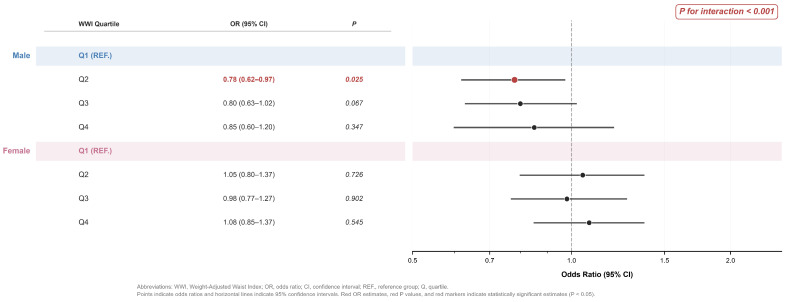
Association of WWI with depressive symptoms stratified by sex. Model adjustments included age in years, residential location, living arrangements, marital condition, educational attainment, and yearly earnings, together with smoking and drinking status, chronic diseases, cognitive function, ADL disability, and vegetable and fruit intake categories.

Multivariable analysis showed that female sex, rural residence, chronic diseases, disability in ADL, and cognitive impairment were significantly associated with depressive symptoms (Table 3). In the total population, the V+/F− category was associated with higher odds of depressive symptoms in both sexes in Model 1. After full adjustment, this association remained significant only in women (AOR = 1.25, 95% CI: 1.04–1.49, *p* = 0.015) (Table 4; Figure 1).

**Figure 2:**
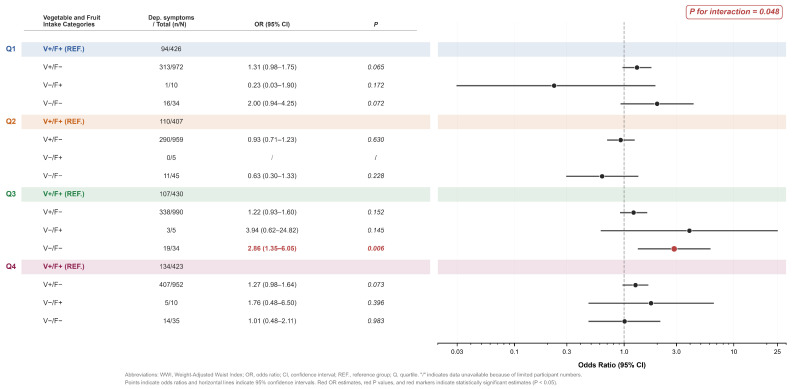
Association of vegetable and fruit intake categories with depressive symptoms stratified by WWI quartiles. Model adjustments included sex, age in years, residential location, living arrangements, marital condition, and educational attainment, together with yearly earnings, smoking and drinking status, as well as chronic diseases, cognitive function, and ADL disability.

An interaction between vegetable and fruit intake categories and WWI levels was observed (*p*_interaction_ = 0.048). In the total population, the V−/F− category was associated with higher odds of depressive symptoms in Q3 (OR = 2.86, 95% CI: 1.35–6.05, *p* = 0.006), whereas associations in the other WWI quartiles were not statistically significant (Figure 2).

### 3.6. Stratified Analyses by WWI Quartiles

Stratified analyses were conducted according to WWI quartiles (Table 4). In the lowest WWI quartile (Q1), significant associations were observed among females; specifically, those with V+/F− (AOR = 2.03, 95% CI: 1.23–3.36, *p* = 0.006) and V−/F− (AOR = 5.12, 95% CI: 1.15–22.81, *p* = 0.032) categories were associated with higher odds of depressive symptoms. In contrast, no significant associations were identified in males within this quartile after multivariable adjustment.

In Q2, no significant associations between vegetable and fruit intake categories and depressive symptoms were observed in either sex after full adjustment (all *p* > 0.05).

In Q3, a significant association was observed among males, with the V−/F− category associated with higher odds of depressive symptoms (AOR = 3.35, 95% CI: 1.22–9.22, *p* = 0.019). Although females in this group exhibited a high point estimate for the V−/F+ category (AOR = 7.27), the association failed to reach statistical significance (*p* = 0.095).

Finally, in the highest WWI quartile (Q4), no significant associations were observed after full adjustment in either sex. Among females, the association between the V+/F− category and depressive symptoms observed in Model 1 (*p* < 0.001) was no longer statistically significant after adjustment (AOR = 1.24, 95% CI: 0.94–1.65, *p* = 0.127).

In summary, statistically significant associations were observed in Q1 females (V+/F− and V−/F−) and in Q3 males (V−/F−) after full adjustment, whereas the majority of WWI- and sex-specific subgroup associations were not statistically significant.

## 4. Discussion

Depressive symptoms were observed in 32.46% of older adults in Anhui Province, exceeding previous national estimates [44]. No significant independent association between WWI and depressive symptoms was observed after multivariable adjustment. Interaction analyses, however, suggested that the association between vegetable and fruit intake categories and depressive symptoms may vary according to metabolic status, as indexed by WWI. This observation differs from previous studies reporting generally lower risks of depressive symptoms among individuals with higher fruit and vegetable intake [45,46].

Among females in the lowest WWI quartile (Q1), the V+/F− category was associated with a higher likelihood of depressive symptoms compared with the V+/F+ category (AOR = 2.03, 95% CI: 1.23–3.36). This observation raises the possibility that vegetables and fruits may contribute differently to mental health among older adults with relatively low metabolic reserves. Vegetables supply fiber, but fruits provide quickly available natural sugars and distinct polyphenols such as anthocyanins that may support neural energy regulation [47,48,49]. Social vulnerability may also contribute: females in Q1 were more likely to live alone and have lower income [50]. Caution is warranted, however, as several subgroup estimates were based on small cell sizes and were accompanied by wide confidence intervals.

Among older males, a significant association was observed only in the Q3 WWI subgroup, where the V−/F− category was associated with higher odds of depressive symptoms (AOR = 3.35, 95% CI: 1.22–9.22). This finding suggests that the association between vegetable and fruit intake categories and depressive symptoms may differ across levels of central adiposity. Previous studies have proposed that central adiposity may be linked to metabolic and inflammatory processes relevant to mental health [32,35]. However, the present data do not allow conclusions regarding the mechanisms involved, and this subgroup-specific finding should be interpreted cautiously.

At the highest WWI level (Q4), no significant associations between vegetable and fruit intake categories and depressive symptoms were observed after adjustment in either sex. Individuals in this group had the greatest burden of multimorbidity, ADL disability, and cognitive impairment; among men, cognitive impairment nearly doubled from Q1 to Q4 [29,51]. One possible explanation is that the coexistence of multiple health conditions may reduce the relative contribution of dietary factors to depressive symptoms. However, the underlying mechanisms cannot be determined from the present data. Alternative explanations, including residual confounding and confounding by indication, cannot be excluded.

Beyond subgroup-specific findings, the absence of a significant direct association between WWI and depressive symptoms among men may reflect the complex interplay of behavioral, metabolic, and health-related factors. For example, smoking and alcohol consumption declined across increasing WWI quartiles, a pattern that may be consistent with the sick-quitter phenomenon reported previously [52,53]. However, the underlying mechanisms remain uncertain and warrant further investigation.

Reverse causation is an important consideration in interpreting these findings [54]. Depressive symptoms may influence dietary behaviors, appetite, food preferences, physical activity, and body weight, thereby affecting both vegetable and fruit intake categories and WWI. Because the relationship among diet, body composition, and mental health is likely bidirectional, the temporal direction of these associations cannot be determined in a cross-sectional study. Longitudinal studies with repeated measures of diet, body composition, and depressive symptoms are needed to clarify these relationships.

To our knowledge, this is the first study to use WWI to examine the joint associations of central adiposity and vegetable and fruit intake categories with depressive symptoms among Chinese older adults, including those with a normal-weight obesity phenotype. Several limitations should be acknowledged.

First, the cross-sectional design precludes causal inference, and all reported associations should be interpreted as correlational. Second, dietary exposure was assessed using self-reported intake frequency categories without information on portion size, total energy intake, dietary diversity, or nutritional composition. Third, several potentially important confounders—including physical activity, sleep quality, frailty, social isolation, antidepressant use, and dietary supplementation—were unavailable and could not be adjusted for. Many of these factors are associated with both dietary behaviors and depressive symptoms in older adults, and residual confounding may have influenced the observed associations. Fourth, the sample was drawn exclusively from Anhui Province and therefore may not be representative of the broader Chinese older population. Fifth, pooled WWI quartiles produced substantial sex imbalances across quartiles, which may have affected the comparability of sex-stratified analyses. Finally, multiple subgroup and interaction analyses were conducted without formal correction for multiple testing. The observed interaction effect was borderline significant (*p*_interaction_ = 0.048) and should be interpreted cautiously, as several subgroup estimates were based on sparse data and very small numbers of participants and depressive cases, resulting in limited precision and wide confidence intervals. These limitations may affect the reliability and interpretability of the reported stratified associations. Accordingly, these findings should be considered exploratory and hypothesis-generating rather than confirmatory.

Despite these limitations, our findings carry several implications. They suggest that the relationship between vegetable and fruit intake and depressive symptoms may depend on metabolic status and sex, underscoring the need to consider population heterogeneity in future work. Dietary intake and body composition may require joint evaluation when examining depressive symptoms in older adults. Incorporating simple measures of central adiposity alongside dietary assessments into geriatric health evaluations could help identify subgroups warranting closer mental health follow-up.

## 5. Conclusions

Our findings suggest that the associations between vegetable and fruit intake categories and depressive symptoms may vary according to WWI level and sex among Chinese older adults. No significant independent association between WWI and depressive symptoms was observed after multivariable adjustment. However, the interaction analyses indicate that metabolic status, as indexed by WWI, may influence the association between dietary intake categories and depressive symptoms. In particular, the observed associations appeared more evident among females in the lowest WWI quartile and males in the third WWI quartile, whereas such associations were less apparent among individuals in the highest WWI quartile.

## Figures and Tables

**Table 1:** Characteristics of 2621 Male Participants by WWI Quartile.

Characteristics	Total (*N* = 2621)	WWI Q1 (*N* = 983)	WWI Q2 (*N* = 809)	WWI Q3 (*N* = 617)	WWI Q4 (*N* = 212)	*X* ^2^	*p*
Age in years						89.25	<0.001
60–69	1229 (46.89)	543 (55.24)	369 (45.61)	250 (40.52)	67 (31.60)		
70–79	1049 (40.02)	355 (36.11)	342 (42.27)	264 (42.79)	88 (41.51)		
≥80	343 (13.09)	85 (8.65)	98 (12.11)	103 (16.69)	57 (26.89)		
Residential location						13.91	0.003
Urban	1248 (47.62)	428 (43.54)	421 (52.04)	303 (49.11)	96 (45.28)		
Rural	1373 (52.38)	555 (56.46)	388 (47.96)	314 (50.89)	116 (54.72)		
Living arrangements						4.11	0.249
Living alone	418 (15.95)	153 (15.56)	118 (14.59)	105 (17.02)	42 (19.81)		
Living with others	2203 (84.05)	830 (84.44)	691 (85.41)	512 (82.98)	170 (80.19)		
Marital condition						7.59	0.055
Married	2065 (78.79)	784 (79.76)	649 (80.22)	479 (77.63)	153 (72.17)		
Other	556 (21.21)	199 (20.24)	160 (19.78)	138 (22.37)	59 (27.83)		
Educational attainment						4.94	0.551
No formal schooling	827 (31.55)	304 (30.93)	256 (31.64)	195 (31.60)	72 (33.96)		
Primary education	939 (35.83)	353 (35.91)	284 (35.11)	217 (35.17)	85 (40.09)		
Higher	855 (32.62)	326 (33.16)	269 (33.25)	205 (33.23)	55 (25.94)		
Yearly earnings (RMB)						11.96	0.008
<6500	1384 (52.80)	507 (51.58)	405 (50.06)	340 (55.11)	132 (62.26)		
≥6500	1237 (47.20)	476 (48.42)	404 (49.94)	277 (44.89)	80 (37.74)		
Smoking status						10.04	0.018
Yes	1054 (40.21)	432 (43.95)	316 (39.06)	230 (37.28)	76 (35.85)		
No	1567 (59.79)	551 (56.05)	493 (60.94)	387 (62.72)	136 (64.15)		
Drinking status						11.12	0.011
Yes	1577 (60.17)	597 (60.73)	513 (63.41)	357 (57.86)	110 (51.89)		
No	1044 (39.83)	386 (39.27)	296 (36.59)	260 (42.14)	102 (48.11)		
Chronic disease						26.18	<0.001
No	799 (30.48)	343 (34.89)	257 (31.77)	150 (24.31)	49 (23.11)		
Yes	1822 (69.52)	640 (65.11)	552 (68.23)	467 (75.69)	163 (76.89)		
Disability in ADL						18.14	<0.001
No	1169 (44.60)	464 (47.20)	371 (45.86)	267 (43.27)	67 (31.60)		
Yes	1452 (55.40)	519 (52.80)	438 (54.14)	350 (56.73)	145 (68.40)		
Cognitive impairment						45.47	<0.001
No	1944 (74.17)	760 (77.31)	617 (76.27)	449 (72.77)	118 (55.66)		
Yes	677 (25.83)	223 (22.69)	192 (23.73)	168 (27.23)	94 (44.34)		
Depressive symptoms						6.22	0.102
Yes	678 (25.87)	273 (27.77)	188 (23.24)	155 (25.12)	62 (29.25)		
No	1943 (74.13)	710 (72.23)	621 (76.76)	462 (74.88)	150 (70.75)		
Vegetable and Fruit Intake Categories						10.06	0.346
V+/F+	755 (28.81)	266 (27.06)	226 (27.94)	192 (31.12)	71 (33.49)		
V+/F−	1774 (67.68)	686 (69.79)	552 (68.23)	404 (65.48)	132 (62.26)		
V−/F+	14 (0.53)	7 (0.71)	5 (0.62)	1 (0.16)	1 (0.47)		
V−/F−	78 (2.98)	24 (2.44)	26 (3.21)	20 (3.24)	8 (3.77)		

**Table 2:** Characteristics of 3116 Female Participants by WWI Quartile.

Characteristics	Total (*N* = 3116)	WWI Q1 (*N* = 459)	WWI Q2 (*N* = 607)	WWI Q3 (*N* = 842)	WWI Q4 (*N* = 1208)	*X* ^2^	*p*
Age in years						188.24	<0.001
60–69	1486 (47.69)	289 (62.96)	362 (59.64)	419 (49.76)	416 (34.44)		
70–79	1214 (38.96)	142 (30.94)	200 (32.95)	322 (38.24)	550 (45.53)		
≥80	416 (13.35)	28 (6.10)	45 (7.41)	101 (12.00)	242 (20.03)		
Residential location						4.75	0.191
Urban	1586 (50.90)	245 (53.38)	315 (51.89)	440 (52.26)	586 (48.51)		
Rural	1530 (49.10)	214 (46.62)	292 (48.11)	402 (47.74)	622 (51.49)		
Living arrangements						15.10	0.002
Living alone	623 (19.99)	65 (14.16)	122 (20.10)	163 (19.36)	273 (22.60)		
Living with others	2493 (80.01)	394 (85.84)	485 (79.90)	679 (80.64)	935 (77.40)		
Marital condition						30.44	<0.001
Married	2061 (66.14)	342 (74.51)	415 (68.37)	567 (67.34)	737 (61.01)		
Other	1055 (33.86)	117 (25.49)	192 (31.63)	275 (32.66)	471 (38.99)		
Educational attainment						54.85	<0.001
No formal schooling	1988 (63.80)	270 (58.82)	348 (57.33)	551 (65.44)	819 (67.80)		
Primary education	665 (21.34)	82 (17.86)	156 (25.70)	169 (20.07)	258 (21.36)		
Higher	463 (14.86)	107 (23.31)	103 (16.97)	122 (14.49)	131 (10.84)		
Yearly earnings (RMB)						24.44	<0.001
<6500	2051 (65.82)	260 (56.64)	393 (64.74)	560 (66.51)	838 (69.37)		
≥6500	1065 (34.18)	199 (43.36)	214 (35.26)	282 (33.49)	370 (30.63)		
Smoking status						1.73	0.631
Yes	148 (4.75)	19 (4.14)	30 (4.94)	46 (5.46)	53 (4.39)		
No	2968 (95.25)	440 (95.86)	577 (95.06)	796 (94.54)	1155 (95.61)		
Drinking status						4.71	0.194
Yes	644 (20.67)	104 (22.66)	139 (22.90)	169 (20.07)	232 (19.21)		
No	2472 (79.33)	355 (77.34)	468 (77.10)	673 (79.93)	976 (80.79)		
Chronic disease						37.05	<0.001
No	849 (27.25)	174 (37.91)	178 (29.32)	207 (24.58)	290 (24.01)		
Yes	2267 (72.75)	285 (62.09)	429 (70.68)	635 (75.42)	918 (75.99)		
Disability in ADL						82.11	<0.001
No	1221 (39.18)	246 (53.59)	277 (45.63)	319 (37.89)	379 (31.37)		
Yes	1895 (60.82)	213 (46.41)	330 (54.37)	523 (62.11)	829 (68.63)		
Cognitive impairment						31.27	<0.001
No	1995 (64.02)	324 (70.59)	405 (66.72)	563 (66.86)	703 (58.20)		
Yes	1121 (35.98)	135 (29.41)	202 (33.28)	279 (33.14)	505 (41.80)		
Depressive symptoms						11.14	0.011
Yes	1184 (38.00)	151 (32.90)	223 (36.74)	312 (37.05)	498 (41.23)		
No	1932 (62.00)	308 (67.10)	384 (63.26)	530 (62.95)	710 (58.77)		
Vegetable and Fruit Intake Categories						15.16	0.087
V+/F+	931 (29.88)	160 (34.86)	181 (29.82)	238 (28.27)	352 (29.14)		
V+/F−	2099 (67.36)	286 (62.31)	407 (67.05)	586 (69.60)	820 (67.88)		
V−/F+	16 (0.51)	3 (0.65)	0 (0.00)	4 (0.48)	9 (0.75)		
V−/F−	70 (2.25)	10 (2.18)	19 (3.13)	14 (1.66)	27 (2.24)		

**Table 3:** Associations of Vegetable and Fruit Intake Categories, WWI, and Other Covariates with Depressive Symptoms.

Variables	Depressive Symptoms		OR, 95% CI	*p*
	No	Yes		
Sex				
Male (REF.)	1943 (74.13)	678 (25.87)		
Female	1932 (62.00)	1184 (38.00)	1.41 (1.21–1.64)	<0.001
Age in years				
60–69 (REF.)	1891 (69.65)	824 (30.35)		
70–79	1508 (66.64)	755 (33.36)	1.03 (0.90–1.17)	0.679
≥80	476 (62.71)	283 (37.29)	1.01 (0.83–1.22)	0.928
Residential location				
Urban (REF.)	2083 (73.50)	751 (26.50)		
Rural	1792 (61.73)	1111 (38.27)	1.35 (1.18–1.53)	<0.001
Living arrangements				
Living with others (REF.)	3225 (68.68)	1471 (31.32)		
Living alone	650 (62.44)	391 (37.56)	1.11 (0.92–1.33)	0.265
Marital condition				
Other (REF.)	1015 (63.00)	596 (37.00)		
Married	2860 (69.32)	1266 (30.68)	0.98 (0.83–1.15)	0.759
Educational attainment				
No formal schooling (REF.)	1686 (59.89)	1129 (40.11)		
Primary education	1132 (70.57)	472 (29.43)	0.79 (0.69–0.92)	<0.001
Higher	1057 (80.20)	261 (19.80)	0.58 (0.49–0.69)	<0.001
Yearly earnings (RMB)				
<6500 (REF.)	2135 (62.15)	1300 (37.85)		
≥6500	1740 (75.59)	562 (24.41)	0.87 (0.76–1.00)	0.047
Smoking status				
No (REF.)	2992 (65.98)	1543 (34.02)		
Yes	883 (73.46)	319 (26.54)	0.93 (0.79–1.10)	0.382
Drinking status				
No (REF.)	2281 (64.87)	1235 (35.13)		
Yes	1594 (71.77)	627 (28.23)	0.96 (0.84–1.10)	0.585
Chronic disease				
No (REF.)	1250 (75.84)	398 (24.15)		
Yes	2625 (64.20)	1464 (35.80)	1.68 (1.47–1.92)	<0.001
Disability in ADL				
No (REF.)	1791 (74.94)	599 (25.06)		
Yes	2084 (62.26)	1263 (37.74)	1.43 (1.26–1.62)	<0.001
Cognitive impairment				
No (REF.)	2828 (71.79)	1111 (28.21)		
Yes	1047 (58.23)	751 (41.77)	1.41 (1.24–1.60)	<0.001
Vegetable and Fruit Intake Categories				
V+/F+ (REF.)	1241 (73.61)	445 (26.39)		
V+/F−	2525 (65.19)	1348 (34.81)	1.19 (1.04–1.36)	0.012
V−/F+	21 (70.00)	9 (30.00)	0.95 (0.42–2.14)	0.907
V−/F−	88 (59.46)	60 (40.54)	1.36 (0.95–1.96)	0.096
WWI				
Q1 (7.27–10.80) (REF.)	1018 (70.60)	424 (29.40)		
Q2 (10.80–11.33)	1005 (70.97)	411 (29.03)	0.87 (0.74–1.03)	0.118
Q3 (11.33–11.94)	992 (67.99)	467 (32.01)	0.87 (0.73–1.03)	0.096
Q4 (11.94–16.08)	860 (60.56)	560 (39.44)	0.95 (0.79–1.14)	0.573

Abbreviations: Q, quartile; REF, reference group; OR, odds ratio; 95% CI, 95% confidence interval.

**Table 4:** Associations Between Vegetable and Fruit Intake Categories and Depressive Symptoms According to Sex and WWI Quartiles.

Participant Group/Intake Categories	Dep. Symptoms /Total (*n*/*N*)	Model 1		Model 2	
		OR, 95% CI	*p*	AOR, 95% CI	*p*
Total Participants (*N* = 5737)					
Male					
V+/F+ (REF.)	164/755				
V+/F−	489/1774	1.37 (1.12–1.68)	0.002	1.13 (0.91–1.39)	0.282
V−/F+	1/14	0.28 (0.04–2.13)	0.218	0.20 (0.02–1.56)	0.123
V−/F−	24/78	1.60 (0.96–2.67)	0.071	1.19 (0.70–2.02)	0.522
Female					
V+/F+ (REF.)	281/931				
V+/F−	859/2099	1.60 (1.36–1.89)	<0.001	1.25 (1.04–1.49)	0.015
V−/F+	8/16	2.31 (0.86–6.22)	0.097	1.97 (0.72–5.43)	0.189
V−/F−	36/70	2.45 (1.50–3.99)	<0.001	1.56 (0.93–2.59)	0.089
WWI Q1 (*N* = 1442)					
Male					
V+/F+ (REF.)	60/266				
V+/F−	203/686	1.44 (1.04–2.01)	0.030	1.07 (0.75–1.53)	0.716
V−/F+	1/7	0.57 (0.07–4.85)	0.609	0.24 (0.03–2.25)	0.212
V−/F−	9/24	2.06 (0.86–4.94)	0.105	1.42 (0.56–3.57)	0.459
Female					
V+/F+ (REF.)	34/160				
V+/F−	110/286	2.32 (1.48–3.62)	<0.001	2.03 (1.23–3.36)	0.006
V−/F+	0/3	/	/	/	/
V−/F−	7/10	8.65 (2.12–35.23)	0.003	5.12 (1.15–22.81)	0.032
WWI Q2 (*N* = 1416)					
Male					
V+/F+ (REF.)	49/226				
V+/F−	136/552	1.18 (0.82–1.71)	0.380	1.00 (0.67–1.49)	0.990
V−/F+	0/5	/	/	/	/
V−/F−	3/26	0.47 (0.14–1.64)	0.236	0.38 (0.11–1.35)	0.133
Female					
V+/F+ (REF.)	61/181				
V+/F−	154/407	1.20 (0.83–1.73)	0.337	0.87 (0.58–1.30)	0.496
V−/F+	0/0	/	/	/	/
V−/F−	8/19	1.43 (0.55–3.74)	0.465	0.89 (0.32–2.45)	0.822
WWI Q3 (*N* = 1459)					
Male					
V+/F+ (REF.)	38/192				
V+/F−	106/404	1.44 (0.95–2.19)	0.087	1.21 (0.78–1.89)	0.398
V−/F+	0/1	/	/	/	/
V−/F−	11/20	4.95 (1.92–12.81)	0.001	3.35 (1.22–9.22)	0.019
Female					
V+/F+ (REF.)	69/238				
V+/F−	232/586	1.61 (1.16–2.22)	0.004	1.24 (0.88–1.76)	0.226
V−/F+	3/4	7.35 (0.75–71.87)	0.087	7.27 (0.71–74.37)	0.095
V−/F−	8/14	3.27 (1.09–9.76)	0.034	2.42 (0.75–7.82)	0.140
WWI Q4 (*N* = 1420)					
Male					
V+/F+ (REF.)	17/71				
V+/F−	44/132	1.59 (0.83–3.06)	0.166	1.67 (0.80–3.51)	0.172
V−/F+	0/1	/	/	/	/
V−/F−	1/8	0.45 (0.05–3.96)	0.474	0.25 (0.03–2.48)	0.237
Female					
V+/F+ (REF.)	117/352				
V+/F−	363/820	1.60 (1.23–2.07)	<0.001	1.24 (0.94–1.65)	0.127
V−/F+	5/9	2.51 (0.66–9.53)	0.176	1.95 (0.49–7.81)	0.348
V−/F−	13/27	1.87 (0.85–4.10)	0.121	1.27 (0.56–2.88)	0.575

Note: Model 1: unadjusted; Model 2: controlled for age in years, residential location, living arrangements, marital condition, and educational attainment, alongside yearly earnings, smoking and drinking status, as well as chronic diseases, cognitive function, ADL disability, and vegetable and fruit intake categories, WWI. Abbreviations: AOR, adjusted odds ratio; OR, odds ratio; 95% CI, 95% confidence interval; REF, reference group; /, data unavailable because of limited participant numbers; Q, quartile.

## Data Availability

For confidentiality, the data analyzed in the present study cannot be publicly shared, and will be available by the corresponding author upon reasonable request.

## Abbreviations

The following abbreviations are used in this manuscript:

WWI	Weight-adjusted Waist Index
Q	Quartile
BMI	Body Mass Index
WC	Waist Circumference
WHtR	Waist-to-Height Ratio
AHLS	Anhui Health and Longevity Survey
PHQ-9	Patient Health Questionnaire
MMSE	Mini-Mental State Examination
ADL	Activities of Daily Living
OR	Odds Ratios
AOR	Adjusted Odds Ratios
CI	Confidence Intervals
RMB	Renminbi
